# The S2 Glycoprotein Subunit Determines Intestinal Tropism in Infectious Bronchitis Virus

**DOI:** 10.3390/microorganisms13081918

**Published:** 2025-08-17

**Authors:** Zhenkai Dai, Jing Zhang, Ying Huang, Benli Huang, Zhengzhong Xiao, Keyu Feng, Guanming Shao, Xinheng Zhang, Qingmei Xie

**Affiliations:** 1 State Key Laboratory of Swine and Poultry Breeding Industry & Guangdong Laboratory for Lingnan Modern Agriculture, College of Animal Science, South China Agricultural University, Guangzhou 510642, China; zhenkai.dai@uqconnect.edu.au (Z.D.);; 2 School of Biology and Agriculture, Shaoguan University, Shaoguan 512005, China; 3 Guangdong Provincial Key Lab of AgroAnimal Genomics and Molecular Breeding, College of Animal Science, South China Agricultural University, Guangzhou 510642, China; 4 Guangdong Engineering Research Center for Vector Vaccine of Animal Virus, Guangzhou 510642, China; 5 School of Education, Arts, Design & Architecture, University of New South Wales, Sydney 2052, Australia; 6 Chuxiong Vocational High School, Chuxiong 675000, China

**Keywords:** avian coronavirus, tissue tropism, spike protein, S2 subunit, poultry disease, viral pathogenesis, duodenum

## Abstract

The molecular basis for the distinct intestinal tropism of infectious bronchitis virus (IBV) strains remains poorly understood. This study identifies the S2 subunit of the spike glycoprotein as the critical determinant conferring duodenal tropism to the IBV CSL strain. Comparative pathogenesis in specific-pathogen-free (SPF) chicks revealed that the CSL strain achieved significantly higher viral titers in the duodenum compared to strains D90, PYG QX1, and XXX QX5. This duodenal replication was associated with severe epithelial inflammation, characterized by upregulation of pro-inflammatory cytokines (IL-6, IL-17A, IL-22, TNF-α, IFN-β, IFN-γ) and disruption of barrier integrity via downregulation of tight junction proteins (Occludin, Claudin-1, ZO-1). Crucially, reverse genetics using the non-enterotropic D90 backbone demonstrated that recombinant viruses carrying the CSL-S2 gene (rD90-ΔS/CSL and rD90-ΔS2/CSL), but not those carrying CSL-S1 (rD90-ΔS1/CSL), replicated efficiently and induced inflammation in the duodenum, phenocopying wild-type CSL. In contrast, renal tropism was independent of the S2 subunit. These findings establish the S2 subunit as both necessary and sufficient for IBV duodenal tropism, uncoupling it from renal pathogenicity. This identifies S2 as a prime molecular target for developing next-generation vaccines against intestinal IBV pathotypes.

## 1. Introduction

Infectious bronchitis virus (IBV), a gammacoronavirus of significant economic consequence, imposes substantial burdens on global poultry production through respiratory disease, nephritis, and enteric pathology [1,2]. IBV is classified within the Coronaviridae family, subfamily Orthocoronavirinae, and genus Gammacoronavirus of the order Nidovirales. This virus possesses a single-stranded, positive-sense RNA genome approximately 27.6 kilobases in length. Characteristic features include a 5′ cap structure and a 3′ polyadenylate tail. The IBV genome contains more than ten identifiable open reading frames (ORFs) [3]. Notably, the size of these ORFs varies across different IBV genotypes. A 7.3 kb segment located near the 3′ terminus encodes the four principal structural proteins: spike (S), envelope (E), membrane (M), and nucleocapsid (N) [3]. The S protein, projecting from the viral envelope as surface projections, is synthesized by the host cell and subsequently cleaved by proteases into the N-terminal S1 and C-terminal S2 subunits. According to the S1 region, IBV exhibits extensive genetic diversity classified into six principal genotypes (GI-GVI) and 32 distinct viral lineages [4]. While the original classification assigned twenty-seven lineages to GI and one lineage to each of the other genotypes, ongoing studies have documented an increase in viral diversity. This includes the identification of additional Genotype 1 lineages (GI-28 [5], GI-29 [6], GI-30 [7]) and the advent of a new genotype designated GVII (GVII-1) [8]. In China, the majority of commonly isolated strains belong to Genotype I (GI). For instance, the QX-type strain falls under the GI-19 lineage. QX-type strains (e.g., PYG QX1, XXX QX5) are frequently utilized as the backbone for live attenuated vaccine vectors due to their high adaptability to embryonated eggs [9,10].

Central to IBV tropism is the trimeric spike (S) glycoprotein, a critical mediator of host cell entry [11,12]. Following post-translational cleavage, the S1 subunit engages host receptors (primarily sialic acids or undefined molecules) [4,13], while the S2 subunit executes membrane fusion [14]. Extensive research confirms that hypervariable regions within S1 dictate receptor specificity, host range, and tissue tropism for respiratory and renal tissues across IBV strains and serotypes [15]. Specific S1 sequences directly determine efficient viral replication in tracheal and renal tissues [16,17]. During viral adsorption to host cells, the S2 glycoprotein serves as a molecular anchor that mediates membrane fusion and facilitates viral entry [18]. Contrasting with the hypervariable S1 subunit, S2 demonstrates higher evolutionary conservation and elicits robust cross-protective immunity. Notably, the proteolytic cleavage site within the S2 subunit of the Beaudette strain regulates membrane fusion efficiency and syncytium formation in Vero cells, indicating its contribution to viral cellular tropism determination [19].

Based on preliminary investigations in this study, CSL strains were discovered and demonstrated strong immunogenicity, making them potential candidate antigen genes for future vaccine development [20]. However, the molecular basis for enteric tropism remains poorly defined. This phenomenon describes the pronounced capacity of specific IBV strains, including the nephropathogenic CSL isolate [20], to undergo robust replication within the duodenum. Understanding this tropism is critical, not only for fundamental virology but also for disease control [21]. Intestinal replication facilitates prolonged viral shedding, enables efficient fecal–oral transmission, potentially exacerbates systemic disease via immune modulation or barrier compromise, and challenges comprehensive vaccine efficacy [22,23].

Given the S protein’s pivotal role in tropism and the established link between S1 variation and other tropisms, the spike glycoprotein is a prime candidate for harboring enteric tropism determinants [18,24]. However, whether S1 or S2 governs this process is unknown. While S1 initiates receptor binding, the S2 subunit orchestrates the complex membrane fusion event, a process potentially fine-tuned to the duodenal microenvironment [25,26]. This unique niche is characterized by dynamic pH gradients and protease abundance, including TMPRSS2 and trypsin, implying S2 may be evolutionarily optimized to leverage such conditions for enhanced entry efficiency [27,28]. Notably, a proteolytic cleavage site within the Beaudette strain’s S2 domain modulates both membrane fusion capacity and syncytia formation in Vero cells, demonstrating this subunit’s direct involvement in determining cellular tropism.

This study aimed to investigate the duodenal pathogenic factors of the IBV CSL strain. Using reverse genetics, we constructed recombinant strains with modified S1 and S2 genes to identify genetic regions responsible for duodenal lesions. Crucially, engineering the non-enterotropic D90 strain to express CSL-S2 conferred specific duodenal replication and pathology, recapitulating wild-type CSL virulence. These findings identify S2 as the primary target for rationally designed vaccines against intestinal IBV.

## 2. Materials and Methods

### 2.1. Virus Strains and Cells

The D90 strains were purchased from Guangdong Dahuanong Animal Health Co., Ltd (Guangzhou, China). The IBV D90 strain and the recombinant strains used in this study were both propagated in 9-day-old specific pathogen-free (SPF) chicken embryos. The BSR-T7/5 cells, a cell line that stably expresses T7 RNA polymerase, were generously provided by Professor Youming Zhang from the Helmholtz Institute of Biotechnology at Shandong University. Chicken kidney (CK) cells were derived from 10-day-old SPF chicken embryos. All these cells were cultured in Dulbecco’s modified Eagle’s medium (DMEM, Gibco, 11966025), supplemented with 10% heat-inactivated fetal bovine serum (FBS, Gibco, A5256701), and 1% antibiotic–antimycotic solution (containing 10,000 I.U./mL of penicillin and 10,000 µg/mL of streptomycin) (Gibco, 15240062). The cells were incubated at 37 °C with 5% CO_2_.

### 2.2. IBV CSL Strain Pathogenicity Test

To preliminarily assess intestinal pathogenicity factors of the CSL strain, 150 one-day-old SPF chicks were randomly allocated to five groups and maintained in separate isolators with free access to feed and water. On day one post-hatch, chicks in four challenge groups were ocularly and nasally inoculated with 10^5.5^EID_50_/0.1 mL of either D90, PYG QX1, XXX QX5, or CSL strains, while controls received equivalent volumes of PBS solution. Chickens were monitored daily for clinical manifestations and mortality. Six birds per group were humanely euthanized for necropsy at 5, 10, and 15 dpi. Tissue specimens, including trachea, lung, kidney, and duodenum, were collected from each bird. Viral loads in these tissues were quantified by qRT-PCR. Surviving birds were monitored for clinical outcomes until 21 dpi.

### 2.3. Plasmid Construction and Viral Rescue

Leveraging our laboratory’s established reverse genetics platform for the D90 strain, we engineered four recombinant viruses by substituting the S, S1, and S2 genes of D90 with their homologs from CSL strains. The resulting constructs—rD90, rD90-ΔS/CSL, rD90-ΔS1/CSL, and rD90-ΔS2/CSL—were successfully rescued and exhibited stable propagation with consistent expression of donor-derived antigenic genes. Gene replacement was performed via Red/ET homologous recombination in E. coli DH10B cells (Thermo Fisher Scientific, Waltham, Massachusetts, USA, EC0113) expressing Redα/Redβ recombinases [29,30]. Commercially synthesized S, S1, and S2 genes from CSL strains (Sangon Biotech, Shanghai, China), flanked by ~40-bp homology arms, were integrated using a two-step strategy: First, “line-loop” recombination in Redα/Redβ-expressing E. coli DH10B gyrA462 inserted the ccdB-amp counter-selection cassette into the expression vector. Second, the ccdB-amp cassette was replaced with target genes (S1 or S2) in modified DH10B cells, enabling selection of correct recombinants through ccdB-mediated negative selection. Recombinant IBV plasmids were co-transfected with pVAX1-D90-N into BSR-T7/5 cells using Lipofectamine^®^ 3000 (Thermo Fisher Scientific, L3000015) per manufacturer guidelines. After 4 h at 37 °C, supernatants were discarded, cells were washed twice with PBS, and fresh DMEM (2% FBS + 1% antibiotics) was added for 72 h. Cell lysates from three freeze–thaw cycles were inoculated into 9-day-old SPF chicken embryos. Allantoic fluid was harvested 48 h post-inoculation, followed by two blind passages in embryos prior to final collection. Viral RNA was extracted from 200 μL aliquots of each recombinant strain using the Novizan Viral Genomic RNA Extraction Kit (Vazyme, Nanjing, China, RC323-01). Full-length S gene expression was verified by RT-PCR with primers D90-S-F (5′-AGTGTGGTAAGTTACTGGTAAG-3′) and D90-S-R (5′-GGACGTGGGACTTTGGATCA-3′), designed against the 3′ terminus of IBV D90 1ab and the 5′ terminus of 3a. The thermal profile was 50 °C for 30 min (reverse transcription); 94°C for 4 min; 32 cycles of 94 °C for 30 s, 55 °C for 45 s, and 72 °C for 1 min 20 s; 72 °C for 5 min; and a 4 °C hold. Products were resolved on 1% agarose gels, UV-visualized, and confirmed by Sanger sequencing.

### 2.4. Recombinant D90 Strain Pathogenicity Test

In order to preliminarily explore the pathogenic factors of the intestine caused by the CSL strain, 180 one-day-old SPF chickens were randomly divided into six groups (*n* = 30), including a control group, a positive control group (CSL strain), and four recombinant D90 strain experimental groups. These chickens were raised in seven isolator units, with free access to food and water.

At the age of one day, six experimental groups of chickens were inoculated via eye drop with five strains of recombinant D90 virus and the CSL strain. The dose for each inoculation was set at 10^5.5^EID_50_/0.1 mL portions of the production immune dose. After inoculation, the state of the chickens was observed and recorded daily, with a focus on changes associated with gastritis. Main observations included clinical signs such as depression, tracheal rales, breathing with neck outstretched and mouth open, tearing, etc., as well as mortality. At 5, 10, and 15 dpi, six chickens from each group were randomly selected, sacrificed, and dissected for observation. Tissue samples from the duodenum were collected, and nucleic acid was extracted from these tissue samples. Virus loads in the duodenum were detected by qRT-PCR. Surviving birds were monitored for clinical outcomes until 21 dpi.

### 2.5. RNA Extraction and Reverse Transcription

Tissue samples were isolated and immediately snap-frozen in liquid nitrogen. The samples were stored at −80 °C until further use. The tissue samples were thawed and then homogenized using a mechanical homogenizer or a pestle and mortar with ceramic beads. TRIzol (Sangon Biotech, B511311) reagent was added to the homogenized tissue samples, and total RNA was extracted according to the manufacturer’s instructions. The RNA was purified using an RNeasy mini kit (Vazyme, Nanjing, China, RC112) or equivalent, following the manufacturer’s protocol. The RNA was eluted in RNase-free water. The RNA was reverse transcribed into cDNA using a reverse transcription kit (Vazyme, R433), following the manufacturer’s protocol. The cDNA was diluted to a suitable concentration for qPCR analysis.

### 2.6. Viral Load Determination

Viral copy number in tissues was then quantified by SYBR Green-based quantitative PCR (qPCR) using the ChamQ Universal SYBR qPCR Master Mix (Vazyme, Q711) with the primer pair F: 5′-CCTCTAAGGGCTTTTGAG-3′ and R: 5′-GTCACTGTCTATTGTATGTC-3′ targeting the IBV 5′UTR to amplify a 144-bp product. The qPCR reaction was performed in a 25 μL volume containing 12.5 μL of ChamQ Universal SYBR qPCR Master Mix (2×), 1 μL of each primer (10 μM), 2 μL of cDNA template, 1 μL of Enzyme Mix, and 7.5 μL of ddH2O, under the following thermal cycling conditions: initial denaturation at 94 °C for 2 min, followed by 40 cycles of denaturation at 94 °C for 15 s and annealing/extension at 60 °C for 30 s, followed by a final extension at 72 °C for 10 min. For absolute quantification, a standard curve was generated in each qPCR run using serial tenfold dilutions of a positive control plasmid DNA, constructed and quantified as described previously [20].

### 2.7. qRT-PCR Analysis of Gene Expression

Intestinal tissue samples were isolated and immediately snap-frozen in liquid nitrogen. They were stored at −80 °C until further use. The tissue samples were thawed and then homogenized using a mechanical homogenizer or a pestle and mortar with ceramic beads. TRIzol reagent was added to the homogenized tissue samples, and total RNA was extracted according to the manufacturer’s instructions. The RNA was purified using an RNeasy mini kit or equivalent, following the manufacturer’s protocol. The RNA was eluted in RNase-free water. The RNA was reverse transcribed into cDNA using a reverse transcription kit, following the manufacturer’s protocol. The cDNA was diluted to a suitable concentration for qPCR analysis. This cDNA served as the template for detecting the relative gene expression levels. The primers of the genes can be seen in Table 1. GAPDH was designated as the reference gene. The thermal cycling parameters were set as follows: an initial denaturation at 94 °C for 2 min, succeeded by 40 PCR cycles consisting of denaturation at 94 °C for 15 s and annealing/extension at 60 °C for 30 s. The 2^−ΔΔ^CT method was applied to determine the fold change in gene expression.

### 2.8. Statistical Analysis

Data are expressed as means ± standard deviations. All data were checked for normality and homogeneity of variance before performing statistical comparisons. Between-group comparisons were conducted using a one-way analysis of variance in GraphPad Prism 9 software. Differences were considered significant at * *p* < 0.05, ** *p* < 0.01, *** *p* < 0.001, and **** *p* < 0.0001.

### 2.9. Ethics Statement

All experiments were carried out in strict accordance with the recommendations of the Guide for the Care and Use of Laboratory Animals of the National Institutes of Health. The use of animals in this study was approved by the South China Agricultural University Committee of Animal Experiments (approval ID: SYXK2022-0136).

## 3. Results

### 3.1. CSL Strain Exhibits Unique Duodenal and Renal Tropism

**Figure 1:**
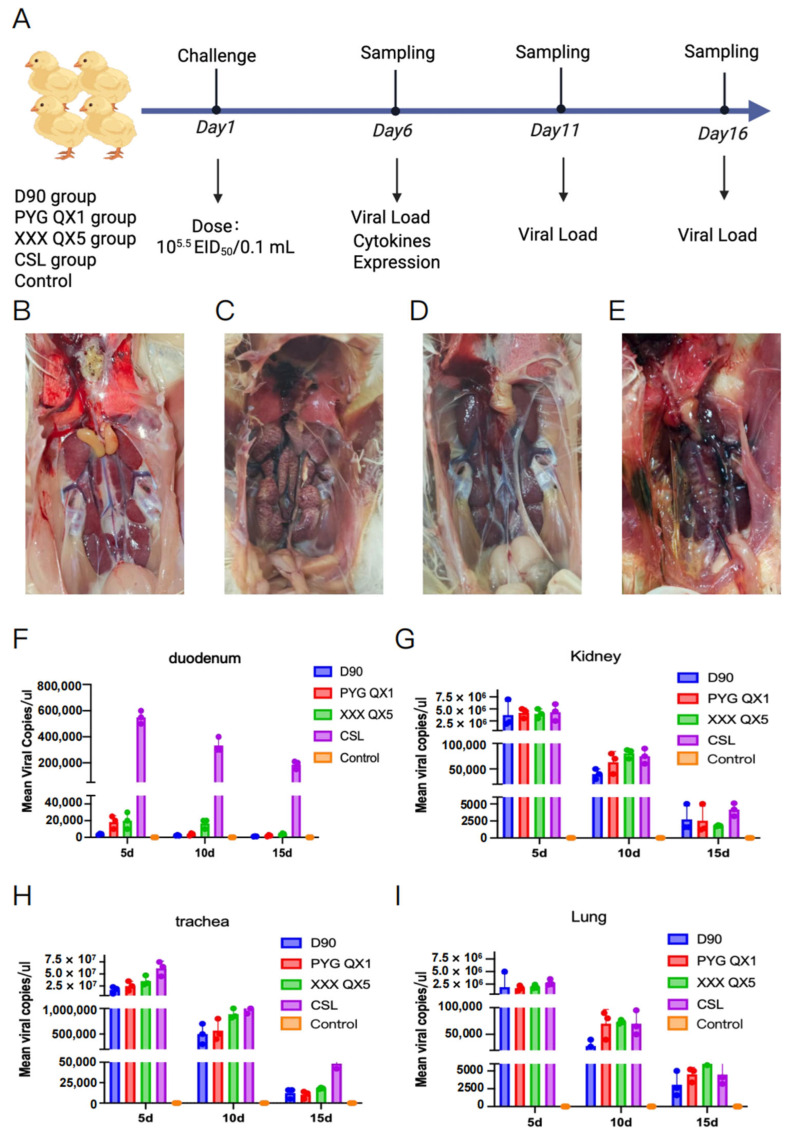
CSL Strain Exhibits Unique Duodenal and Renal Tropism. On day 1 post-hatching, chicks in four challenge groups were intranasally inoculated with 10^5.5^ EID_50_/0.1 mL of virus. At 5, 10, and 15 days post-infection (dpi), six chickens per group were randomly euthanized for duodenal sample collection to quantify viral load. Clinical manifestations were concurrently recorded. (**A**). Schematic diagram illustrating the experimental procedure for the animal experiment. (**B**). Normal anatomy without lesions. (**C**). Mottled kidneys with urate deposits. (**D**). Bilateral renal swelling. (**E**). Bilateral airsacculitis. (**F**–**I**). The graph shows viral RNA copies of the different tissues at 5, 10, and 15 dpi in different groups.

To detect the tropism of the CSL strain, we performed the IBV CSL strain pathogenicity test. On day 1 post-hatch, chicks in four challenge groups (D90, PYG QX1, XXX QX5, and CSL IBV strains) were intranasally inoculated with 10^5.5^ EID_50_/0.1 mL of their respective virus. Duodenal samples were collected from six randomly selected chickens per group at 5, 10, and 15 dpi for viral load assessment (Figure 1A). Figure 1B depicts normal anatomy without lesions. At 10 dpi, chicks challenged with the CSL IBV strain exhibited characteristic gross lesions, including mottled kidneys, urate deposition (Figure 1C), bilateral renal swelling (Figure 1D), and bilateral airsacculitis (Figure 1E).

Viral load analysis demonstrated organ-specific patterns. In the duodenum, the CSL strain exhibited significantly higher viral loads than the other three strains at 5 dpi, 10 dpi, and 15 dpi (Figure 1F). Regarding kidney tissues, the CSL strain maintained significantly higher viral loads than reference strains across all time points (5, 10, and 15 dpi), consistent with its pronounced renal pathology (Figure 1G). Tracheal viral loads showed no significant inter-strain differences at any time point (Figure 1H). Similarly, lung viral loads revealed no significant differences among strains at any time point (Figure 1I). Collectively, these results, combined with clinical observations, indicate that the CSL strain exhibits tropism for replication in the duodenum and kidneys. D90, PYG QX1, and XXX QX5 are nephropathogenic strains with no documented duodenal tropism (Figure S1). Their low duodenal loads likely reflect viremia without replication.

### 3.2. CSL Infection Induces Duodenal Epithelial Inflammation

**Figure 2:**
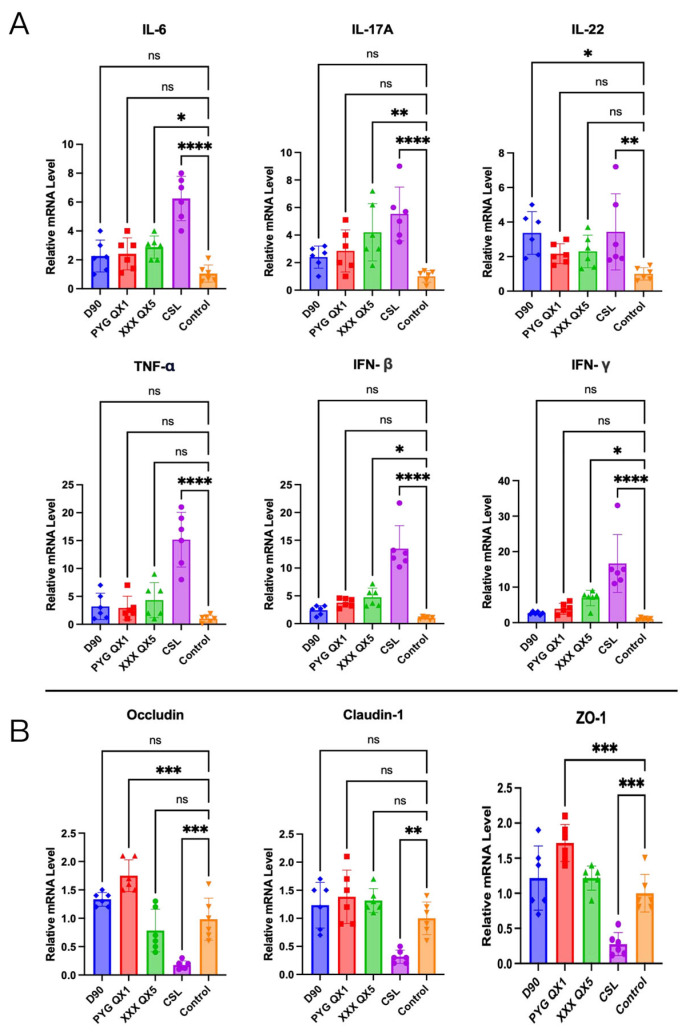
CSL infection induces duodenal epithelial inflammation. RNA samples were extracted from the duodenum tissue of chickens in different groups: D90, PYG QX1, XXX QX5, the CSL-infected group, and the control group at 5 dpi. (**A**). The expression levels of intestinal cytokines, including IL-6, IL-17A, IL-22, IFN-γ, IFN-β, and TNF-α, were determined by qPCR (*n* = 6). (**B**). Additionally, the expression levels of intestinal tight junction proteins, namely Occludin, Claudin-1, and ZO-1, were also quantified using qPCR (*n* = 6). Data are represented as means ± SD. ns stands for not significant, * *p* < 0.05, ** *p* < 0.01, *** *p* < 0.001, and **** *p* < 0.0001.

Viral load analysis indicated peak IBV replication in the duodenum at 5 dpi. Accordingly, two-centimeter duodenal tissue samples were collected from each group at this time point for cytokine mRNA quantification. The qPCR analysis revealed significant upregulation of IL-6, IL-17A, IL-22, IFN-β, IFN-γ, and TNF-α in duodenal epithelial cells following IBV CSL strain infection (Figure 2A). This cytokine overexpression potentially contributes to the strain’s enhanced duodenal viral load and indicates robust inflammatory activation.

Concurrently, we assessed intestinal barrier integrity through tight junction protein expression. The CSL-infected group exhibited significantly reduced mRNA levels of Occludin, Claudin-1, and ZO-1 compared to other strains (Figure 2B), indicating compromised epithelial barrier function.

### 3.3. S2 Subunit Is Necessary and Sufficient for Duodenal Tropism

**Figure 3:**
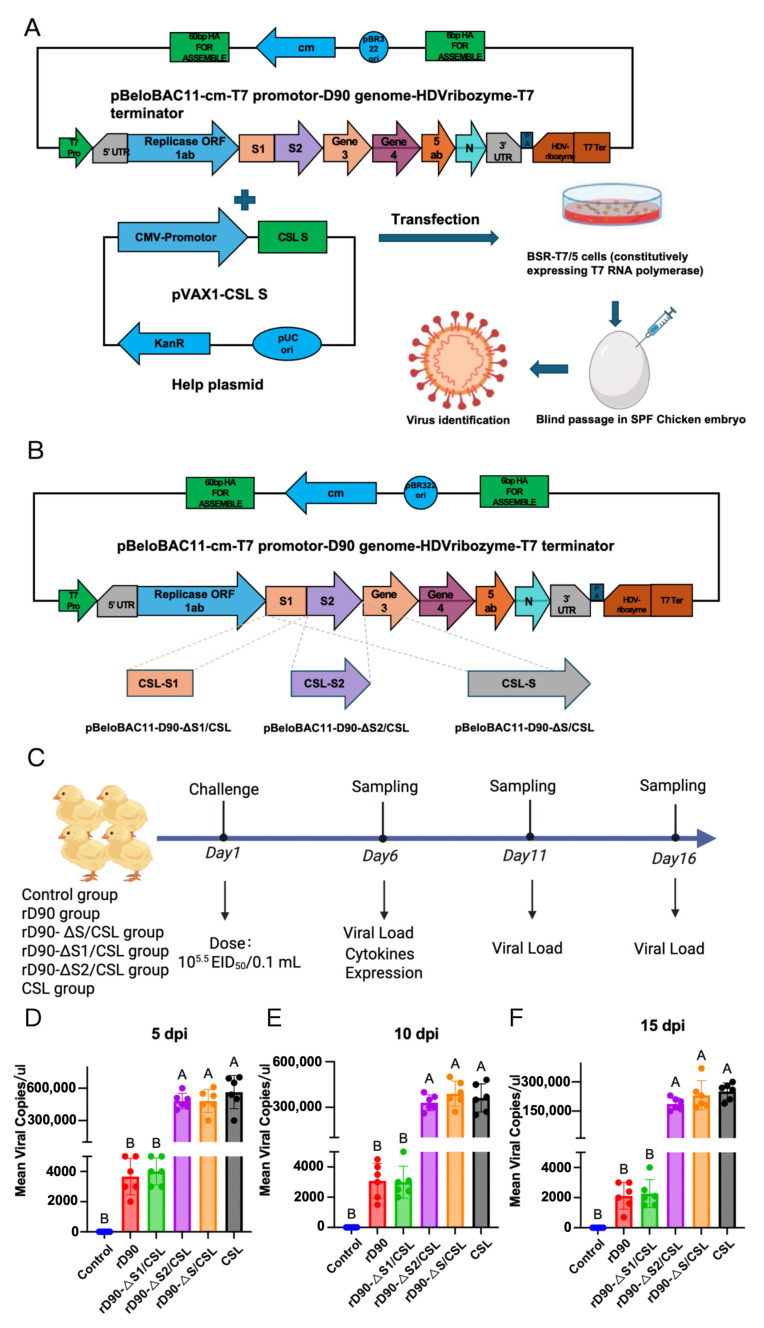
S2 subunit is necessary and sufficient for duodenal tropism. (**A**). Schematic of recombinant infectious clone pBeloBAC11-D90-ΔS/CSL construction. The rD90-ΔS/CSL strain was generated through targeted gene replacement in the parental pBeloBAC11-D90 backbone using a two-step recombination system. Recombinant viruses rD90-ΔS1/CSL and rD90-ΔS2/CSL were constructed following an identical methodology. (**B**). This meticulous procedure yielded four distinct recombinant strains: rD90, rD90-ΔS/CSL, rD90-ΔS1/CSL, and rD90-ΔS2/CSL. (**C**). Schematic diagram illustrating the experimental procedure for the recombinant strains’ infection experiment. (**D**). The mean viral RNA copies of the duodenum at 5 dpi in different groups. (**E**). The mean viral RNA copies of the duodenum at 10 dpi in different groups. (**F**). The mean viral RNA copies of the duodenum at 15 dpi in different groups. Two groups marked with different letters indicate a significant difference (*p* < 0.05). (One-way ANOVA with Tukey’s post hoc comparisons.)

Current research indicates that the S protein primarily governs IBV tissue and cellular tropism. Previous experiments established that strain D90 lacks duodenal pathogenicity. Therefore, the genomic backbone of the D90 strain was selected for replacing the S gene of the CSL strain. Using our laboratory’s reverse genetics platform, we systematically replaced key regions of the CSL S gene to identify genetic determinants of intestinal lesions. This approach enabled successful rescue of four recombinant viruses: parental rD90, rD90-ΔS/CSL, rD90-ΔS1/CSL, and rD90-ΔS2/CSL (Figure 3A,B).

To investigate strain-dependent variations in tissue viral loads, duodenal samples were collected from six randomly selected chickens per group at 5, 10, and 15 dpi for IBV quantification (Figure 3B). As shown in Figure 3C,D, both rD90-ΔS2/CSL and rD90-ΔS/CSL strains exhibited significantly higher duodenal viral loads than the parental rD90 strain at 5 dpi and 10 dpi. By 15 dpi, viral loads in these groups markedly decreased but remained significantly elevated compared to rD90 (Figure 3E). In contrast, duodenal viral loads in rD90-ΔS1/CSL-infected chickens showed no significant difference from rD90 at any time point (5, 10, or 15 dpi) (Figure 3C–E). These results demonstrate that replacement of the S2 gene specifically enhances viral replication in the duodenum.

### 3.4. CSL S2 Subunit Induces Duodenal Epithelial Inflammation

**Figure 4:**
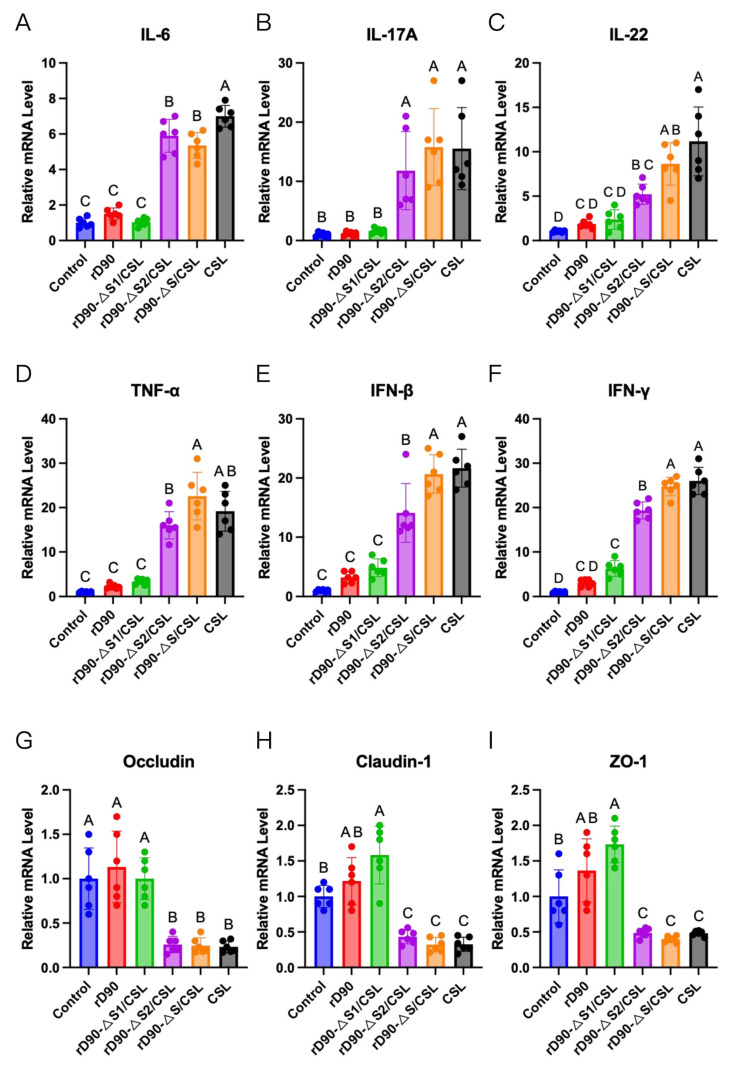
CSL S2 subunit induces duodenal epithelial inflammation. RNA samples were extracted from the duodenum tissue of chickens in different groups: control, rD90, rD90-ΔS/CSL, rD90-ΔS1/CSL, rD90-ΔS2/CSL, and the CSL group at 5 dpi. (**A**–**F**) The expression levels of intestinal cytokines, including IL-6, IL-17A, IL-22, IFN-γ, IFN-β, and TNF-α, were determined by qPCR (n = 6). (**G**–**I**) Additionally, the expression levels of intestinal tight junction proteins, namely Occludin, Claudin-1, and ZO-1, were also quantified using qPCR (n = 6). Two groups marked with different letters indicate a significant difference (*p* < 0.05). (One-way ANOVA with Tukey’s post hoc comparisons.)

To assess duodenal epithelial inflammation induced by the IBV CSL strain, two-centimeter duodenal tissue samples were collected per group at 5 dpi. The qPCR analysis of duodenal epithelial cells revealed significant upregulation of pro-inflammatory cytokines (IL-6, IL-17A, IL-22, IFN-β, IFN-γ, and TNF-α) in rD90-ΔS/CSL and rD90-ΔS2/CSL-infected chickens compared to parental rD90 (Figure 4A–F). In contrast, rD90-ΔS1/CSL-infected chickens showed no significant differences in cytokine expression (Figure 4A–F). Concurrently, tight junction protein expression analysis demonstrated significantly reduced Occludin, Claudin-1, and ZO-1 levels in rD90-ΔS/CSL and rD90-ΔS2/CSL groups compared with rD90 controls (Figure 4G–I). The rD90-ΔS1/CSL group maintained TJ protein expression comparable to rD90 (Figure 4G–I). These findings indicate that the CSL strain’s S2 gene mediates both duodenal epithelial inflammation and barrier dysfunction in chickens.

## 4. Discussion

This study demonstrates that the S2 subunit of the spike glycoprotein is the essential molecular determinant governing the distinctive duodenal tropism of the IBV CSL strain. Our findings provide new insights into understanding coronavirus tissue specificity. While polymorphisms within the hypervariable S1 subunit drive receptor binding and tropism for respiratory tissues in IBV [31,32], we demonstrate that duodenal tropism is uniquely orchestrated by the S2 fusion subunit. Reverse genetics unequivocally showed that only recombinants harboring the CSL-S2 subunit achieved high-titer duodenal replication and phenocopied wild-type CSL. Critically, expression of CSL-S1 alone was insufficient, establishing S2 as both necessary and sufficient for this enteric tropism. This challenges the prevailing view that tissue specificity depends solely on S1–receptor interactions. Future work mapping specific S2 domains—such as the fusion peptide or heptad repeats—will clarify the precise structural basis for duodenal entry.

This study demonstrated significantly higher duodenal viral loads for the CSL strain compared to the other three strains. Crucially, recombinant viruses carrying the CSL-S2 subunit (rD90-ΔS2/CSL and rD90-ΔS/CSL) also exhibited significantly elevated duodenal replication relative to the parental rD90 strain. We therefore propose that the CSL-S2 subunit determines intestinal tropism. Fan et al. constructed S2 locus mutant strains using reverse genetics, which demonstrated significantly divergent syncytium-forming capacities in chick embryonic kidney cells [33]. These findings confirm the crucial role of the S2 subunit in infectious bronchitis virus (IBV) infection, particularly in mediating membrane fusion. We further propose that adaptation of the viral fusion machinery to the duodenal microenvironment underlies intestinal tropism. The intestinal epithelium presents dynamic pH gradients and abundant proteases such as TMPRSS2 or trypsin [34,35]. CSL-S2 likely modulates fusion pH thresholds or exploits local protease activation pathways, facilitating efficient viral entry—a mechanism consistent with S2 adaptations—in enteric coronaviruses like porcine epidemic diarrhea virus (PEDV) [36,37].

Furthermore, CSL-S2-driven replication triggered potent pro-inflammatory cytokines (IL-6, IL-17A, TNF-α, IFN-β, IFN-γ) and disrupted epithelial barrier integrity through reduced Occludin, Claudin-1, and ZO-1 expression. Cytokines are pivotal in orchestrating the host’s immune response to viral infections. Previous studies have shown that pro-inflammatory cytokines, which enhance host resistance to viruses and establish humoral immunity to reduce viral load [38,39], can be counterbalanced by anti-inflammatory cytokines such as IL-6 [39]. However, maintaining a balance between pro- and anti-inflammatory cytokines is essential for mounting an effective defense against viral infection and facilitating tissue repair [40]. This inflammation may initiate a feed-forward loop wherein barrier dysfunction potentiates viral access to basolateral receptors or facilitates spread, thereby amplifying local replication and pathogenesis [41,42].

These findings have profound implications for IBV control. Duodenal tropism underpins prolonged fecal shedding and efficient fecal–oral transmission, key factors in IBV endemicity. Targeting S2 offers a novel avenue to disrupt transmission cycles. Translationally, engineering the non-enterotropic D90 vaccine strain into a duodenum-tropic virus via S2-swapping provides a blueprint for next-generation live-attenuated vaccines. Such chimeras could elicit robust mucosal immunity at intestinal replication sites while leveraging established safe backbones. This approach holds promise against global enteric IBV pathotypes. The broader relevance of S2-driven tropism mechanisms to other enteric coronaviruses warrants exploration for conserved therapeutic targets. Although qRT-PCR quantitatively demonstrates replication differences, complementary immunohistochemical detection of viral proteins would provide stronger evidence for tissue-specific replication, with these methodological constraints warranting guarded interpretation of our experimental pathogenesis model.

In conclusion, we identify the S2 subunit as the molecular switch activating IBV duodenal tropism. This discovery redefines coronavirus tissue specificity paradigms and enables rationally designed vaccines against economically devastating intestinal IBV disease.

## 5. Conclusions

This study establishes the S2 subunit of the spike glycoprotein as the essential molecular determinant governing the duodenal tropism of the IBV CSL strain. Through comparative pathogenesis and reverse genetics, we demonstrate that the S2 subunit is both necessary and sufficient for high-titer viral replication in the duodenum, while renal tropism operates independently. The CSL-S2 subunit drives localized epithelial inflammation via upregulation of pro-inflammatory cytokines (IL-6, IL-17A, IL-22, TNF-α, IFN-β, IFN-γ) and compromises intestinal barrier integrity by suppressing tight junction proteins (Occludin, Claudin-1, ZO-1). Crucially, engineering the non-enterotropic D90 strain with CSL-S2 conferred duodenum-specific replication and pathology, phenocopying wild-type CSL. These findings identify S2 as a prime target for rationally designed vaccines against intestinal IBV pathotypes.

## Figures and Tables

**Table 1:** Primers used for qRT-PCR.

Gene	Primer Sequence (5′–3′)	
	Forward Primer	Reverse Primer
*IL 6*	CCTCCTCGCCAATCTGAAGT	CAAATAGCGAACGGCCCTCA
*IL 22*	CAGGAATCGCACCTACACCT	TCATGTAGCAGCGGTTGTTC
*IL 17A*	CAGGAATCGGTCTCTCGCTC	AGTGAGTTCAAGCAGCCCAA
*IFN γ*	ATCATACTGAGCCAGATTGTTTCG	TCTTTCACCTTCTTCACGCCAT
*TNF α*	CCGCCCAGTTCAGATGAGTT	GCAACAACCAGCTATGCACC
*IFN β*	TCAACATGCTTAGCAGCCCA	AGTGAGTTCAAGCAGCCCAA
*Occludin*	GATGGACAGCATCAACGACC	CATGCGCTTGATGTGGAAGA
*Claudin-3*	GAAGGGCTGTGGATGAACTG	GAGACGATGGTGATCTTGGC
*ZO-1*	GCCTGAATCAAACCCAGCAA	TATGCGGCGGTAAGGATGAT
*GAPDH*	TGAAAGTCGGAGTCAACGGA	GGTCACGCTCCTGGAAGATA

## Data Availability

The original contributions presented in this study are included in the article/supplementary material. Further inquiries can be directed to the corresponding author.

## Supplementary Materials

The following supporting information can be downloaded at: https://www.mdpi.com/article/10.3390/microorganisms13081918/s1, Figure S1: Nephropathogenic potential of D90, PYG QX1, and XXX QX5 strains. A. Gross renal pathology in D90-infected chickens at 10 dpi: bilateral renal enlargement. B. Renal lesions induced by PYG QX1 at 10 dpi: bilateral renal swelling. C. Kidney pathology following XXX QX5 infection at 10 dpi: mottled appearance with diffuse urate deposits.
