# Supplementary material for: The S2 Glycoprotein Subunit Determines Intestinal Tropism in Infectious Bronchitis Virus
Source: Microorganisms. 2025 Aug 17;13(8):1918. doi: 10.3390/microorganisms13081918 (PMC12388379; doi:10.3390/microorganisms13081918)
Supplement: Supplementary file 1 [file microorganisms-13-01918-s001.zip › microorganisms-3777133-supplementary.pdf]

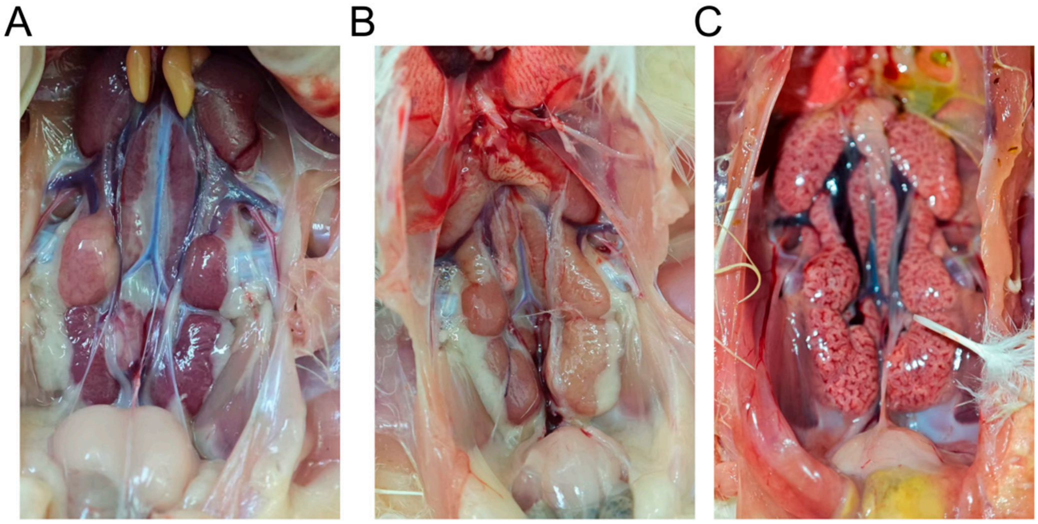

Figure S1. Nephropathogenic potential of D90, PYG QX1, and XXX QX5 strains.

A. Gross renal pathology in D90-infected chickens at 10 dpi: bilateral renal enlargement.

B. Renal lesions induced by PYG QX1 at 10 dpi: bilateral renal swelling.

C. Kidney pathology following XXX QX5 infection at 10 dpi: mottled appearance with diffuse urate deposits.
